# Relationship of Leishmania RNA Virus (LRV) and treatment failure in clinical isolates of *Leishmania major*

**DOI:** 10.1186/s13104-020-04973-y

**Published:** 2020-03-04

**Authors:** Mohsen Abtahi, Gilda Eslami, Serena Cavallero, Mahmood Vakili, Saeedeh Sadat Hosseini, Salman Ahmadian, Mohammad Javad Boozhmehrani, Ali Khamesipour

**Affiliations:** 1 Research Center for Food Hygiene and Safety, Shahid Sadoughi University of Medical Sciences, Shohadaye Gomnam Blv, Yazd, Iran; 2 Department of Parasitology and Mycology, School of Medicine, Shahid Sadoughi University of Medical Sciences, Yazd, Iran; 3 Department of Public Health and Infectious Diseases, Parasitology Section, Sapienza University of Rome, Rome, Italy; 4 Department of Community and Preventive Medicine, Health Monitoring Research Center, School of Medicine, Shahid Sadoughi University of Medical Sciences, Yazd, Iran; 5 Center for Research and Training in Skin Diseases and Leprosy, Tehran University of Medical Sciences, Tehran, Iran

**Keywords:** Cutaneous Leishmaniasis, *Leishmania* RNA Virus, Treatment Failure, *Leishmania major*, SYBR Green Real-Time PCR

## Abstract

**Objective:**

Leishmaniasis is caused by different *Leishmania* spp. Treatment failure (TF) of cutaneous leishmaniasis (CL) is a serious issue that may be due to various reasons, previous studies suggested Leishmania RNA virus (LRV) as a potential cause of TF. Two variant groups of LRV1 and LRV2 are reported. In this study, the presence of LRV1/LRV2 was compared in TF with treatment response (TR) isolates of *L. major*. Clinical isolates of 15 TF and 15 TR were collected from CL patients referred to the Health Centers of Isfahan. Genomic DNA was extracted to identify *Leishmania* spp. using ITS1-PCR–RFLP. Identification of LRV1/LRV2 was performed using SYBR Green Real-Time PCR. The statistical analysis to test relationship between the treatment response with Glucantime and the presence of LRV were performed using SPSS 16.0 with Fisher’s Exact test. *P* value of less than 0.05 was considered significant.

**Results:**

ITS1-PCR–RFLP results showed that every isolate was identified as *L. major*. The results showed no LRV1 in any of the samples but 7 TR isolates and 2 TF isolates showed positive for LRV2. Statistical analysis showed no significant difference between the presence of LRV2 and response to Glucantime (*p*-value = 0.1086). Therefore, other mechanisms might be responsible for TF.

## Introduction

Different species of *Leishmania* cause a wide range of clinical manifestation, including a simple self-healing skin lesion called cutaneous leishmaniasis (CL), a systemic fatal form called visceral leishmaniasis (VL), and mucocutaneous leishmaniasis (MCL). The diseases are transmitted by *Phlebotomus* spp. Most cases of CL occur in Afghanistan, Algeria, Brazil, Colombia, the Islamic Republic of Iran, Peru, Saudi Arabia, and the Syrian Arab Republic [1–3]. The causative agents of CL in the old world are *L. major, L. tropica*, and *L. aethiopica* [4].

Sodium stibogluconate and meglumine antimoniate are the first-line treatment [5]. The occurrence of low response, treatment failure (TF), and resistance are reported from some endemic regions of the world [6]. Different mechanisms have been proposed to explain the lack of response to treatment drugs [7] such as presence of Leishmania RNA virus (LRV) (Family: Totiviridae) which infects–*Leishmania* [8–10]. LRV is a double-stranded RNA (dsRNA), which exists in some *Leishmania* isolates [8–11] and suggested as a virulence factor in some reports [12]. Two species of LRV1 and LRV2 are known [12–14]. So far, no LRV1 has been found in *Leishmania* spp. from new world countries, with the exception of *L. panamensis*, evidence potentially related to the virulence of the parasite. The LRV2 has been reported from the old world human cases of leishmaniasis [8, 15]. LRV2 has also been found in *L. aethiopica* isolated from the biopsy of Ethiopian CL patients [16].

It is reported that the presence of LRV viruses in *Leishmania* spp. might lead to destructive hyper-inflammation resulted in disease severity and parasite metastasis [17]. In addition, previous studies on human cases suggested that LRV in *Leishmania* spp. may exacerbate clinical prognosis of CL, and induce MCL development [18, 19] and possibly TF [14–17].

Despite recent findings of LRV role on *Leishmania* pathogenicity, a few reports exist on TF in old and new world cases of leishmaniasis (16-18). The aim of the present study was to study the presence of Leishmania RNA virus in human cases of CL caused by *L. major* and compare the rate of TF to treatment response.

## Main text

### Ethical consideration

This study was approved by Ethics Committee of Shahid Sadoughi University of Medical Sciences, Yazd, Iran. The informed consent was signed by each patient recruited based on Helsinki declaration.

### Study population

The analytical cross-sectional study included a population of patients, with zoonotic CL/caused by *L. major*, who were referred to the Health Centers of Isfahan, Iran, from September 2017 to December 2018. All cases agreed to participate and signed an informed consent form. Patients who discontinued treatment with Glucantime were excluded. *Leishmania* parasites have been isolated from two population of CL patients who responded to the treatment referred to as treatment response (TR) and patients not responded to treatment as treatment failure (TF); the first category includes 15 isolates from the patients with lesion(s) cured after one full course of treatment, the second group consists of 15 isolates collected from the patients who had active lesion(s), after three courses of treatment with Glucantime (20 mg/kg/day for 14 days in each course) and were considered as TF.

### Sampling

Samples were collected by scrubbing the lesion edge after disinfecting by using 70% alcohol. About 10 mg of each sample was transferred on a slide for direct microscopic examination and subsequent DNA extraction in order to detect and identify *Leishmania* spp. About 10 mg of each sample was transferred into RNA *later* and stored at − 20 °C for search for the presence of *Leishmania* RNA virus (LRV1 and LRV2).

### Microscopic examination

The smear was fixed using methyl alcohol (methanol), then, the slide was stained using Giemsa. Microscopic examination was carried out using 100 × magnifications to find the Leishman bodies. The slides with positive Leishman body were used for molecular identification.

### DNA extraction

DNA was isolated from *Leishmania* positive smears using DNA extraction kit (GenAll, South Korea) based on manufacturer’s instructions. The quality and quantity of the isolated DNA were evaluated using agarose gel electrophoresis and spectrophotometer using nanodrop (ABI, USA).

### Detection and identification

To detect and identify *Leishmania* spp., a diagnostic key based on restriction banding pattern of ITS1 was used based on specific primer pair of LITSr-F 5′-CTGGATCATTTTCCgATg-3′ and L5.8 s-R 5′-TGATACCACTTATCGCACTT-3′ [20]. Amplification reaction mixture included 1x PCR buffer, 0.2 mM dNTP, 1.5 mM MgCl_2_, 1.5 U *Taq* DNA polymerase, 0.5 µM each primer pair, and 100 ng DNA. The amplification protocol was: 35 cycles of 94 °C for 45 s, 50 °C for 45 s, and 72 °C for 45 s. The final extension was done by 72 °C for 5 min. Positive and negative control with DNA of *L. major* (MRHO/IR/75/ER) and ddH_2_O, respectively, were applied in each amplification reaction. The amplification analysis was done using agarose gel electrophoresis (1%) alongside with 50 bp DNA ladder and was visualized by gel documentation system (ATP, Iran, Tehran, G940401). The expected amplicon was about 300–50 bp for detection of *Leishmania* genus.

Molecular identification was performed on positive amplicons digested using *Hae* III (*Bsu* RI) for 3 h at 37 °C. The digestion banding pattern analysis was assessed using agarose gel electrophoresis 3% alongside with 50 bp DNA ladder and *L major* (MRHO/IR/75/ER) as positive control. A banding pattern was visualized using gel documentation system revealing two main bands of 220 and 140 bp pattern considered as *L. major*. All tests were done in triplicate.

The patients with *L. major* were followed for 3 months. The cases with no responses to Glucantime treatment were considered as TF and the cases with complete cure were considered as TR.

### RNA extraction

Total RNA extraction was performed using the Gf-1 Total RNA extraction kit (Vivantis, South Korea) according to the manufacture’s instruction. The quality and quantity of the isolated RNA were checked using agarose gel electrophoresis (1%) and spectrophotometer by nanodrop (ABI, USA).

### cDNA synthesis

Synthesis of cDNA was obtained using cDNA synthesis kit (Fermentas, USA) in accordance with the manufacture’s instruction. The cDNA was amplified using specific primer pair of kmp11-F 5′-GCC TGG ATG AGG AGT TCA ACA-3′ and kmp-11-R 5′-GTC CTC CTT CAT CTC GGG-3′ [21]. The reaction was performed by a mixture of 1x PCR buffer, 0.2 mM dNTP, 1.5 mM MgCl_2_, 1.5 U *Taq* DNA polymerase, and 0.5 µM each primer pair of kmp11. The reaction protocol was done by 40 cycles of 94 °C for 45 s, 60 °C for 45 s, and 72 °C for 45 s. The final extension was done by 72 °C for 5 min. Positive and negative control was applied in each amplification by *L. major* (MRHO/IR/75/ER) and ddH_2_O. The amplification analysis was realized with agarose gel electrophoresis (3%) alongside with positive control (*L. major* MRHO/IR/75/ER), negative control (ddH_2_O), and 50 bp DNA ladder. The experiments were done triplicate.

### Detection of LRV1/LRV2

The presence of LRV1 performed using SYBR Green Real-Time PCR by two sets of specific primers of LRV1-setA-F 5′-CTG ACT GGA CGG GGG GTA AT-3′ and LRV1-setA-R 5′-CAA AAC ACT CCC TTA CGC-3′, and LRV1-setB-F 5′-GTC TGT TTC GTA CCC GCC G-3′ and LRV1-setB-R 5′-AAG CTC AGG ATG TGC ATG TTC CA-3′ [21]. For LRV2 detection the specific primer pair of LRV2-F 5′-GCC ATT ACC CAG CCA GCC AT-3′ and LRV2-R 5′-GCC GTC ACC AGC TCT GTT GT-3′ (this study). The Kmp11 was considered as endogenous control with the primer pair of 5′-GCC TGG ATG AGG AGT TCA ACA-3′ and 5′-GTG CTC.

CTT CAT CTC GGG-3′ [19]. The reaction mixture for all primer pairs was done in 20 µl volume, including 200 nM each primer pair. The reaction temperature was 95 °C for 5 min in one cycle, followed by 40 cycles of 95 °C for 10 s and 60 °C for 1 min in order to annealing and extension. The melting curve was designed after the mentioned cycle.

### Statistical analysis

The data was statistically analyzed using SPSS 16.0 by Fisher’s Exact test for possible relationship between the response to Glucantime treatment and the presence of LRV (LRV1 and LRV2).

### Results

#### Patients

**Fig. 1:**
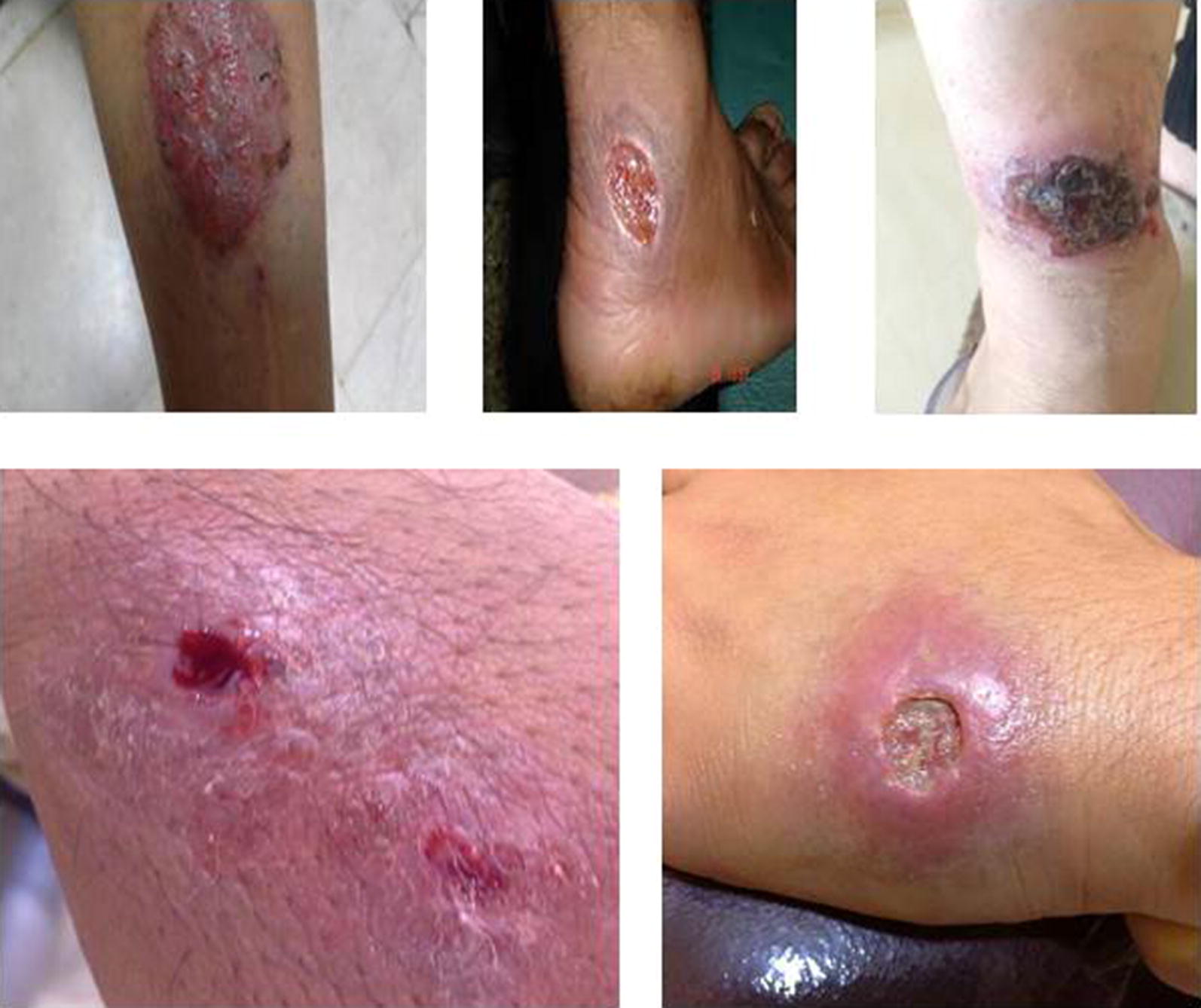
Lesions from patients harboring zoonotic cutaneous leishmaniasis with treatment failure to Glucantime

Upon the results pf PCR, only the *L. major* isolates were included in this study. A total of 30 isolates, 15 TF (Fig. 1) and 15 TR isolates were included in the study. The number of lesions in the TF and TR isolates was approximately the same with an average of 2. The mean age of the patients was 26 years (M = 23, F = 31 years). There was no significant difference between the age of the patients with TR isolates (25 years) and TR isolates (26 years).

#### Molecular diagnosis and identification

The results of molecular detection confirmed that 30 isolates were positive for *Leishmania* and the species identification showed all 30 isolates belonging to *L. major*. The expected size of PCR amplicons was around350 bp (Additional file 1: Figure S1). The expected size of the bands after enzymatic digestion was of 127 and 220 bp (Additional file 2: Figure S2).

#### Assessing of the extracted RNA

The mean of 260/280 ratio at spectrophotometer analyses the extracted RNA was 1.98 ± 0.2 with the mean concentration of 136.2 ± 7.45 ng/μl. After extraction of RNA and synthesis of cDNA, in order to control the system for amplifiability, PCR was done using the specific primer pair of kmp11. All the synthesized cDNA were successfully amplified.

#### Present of LRV1/LRV2

The presence of LRV1 and LRV2 were checked using specific primer pairs using SYBR Green Real-Time PCR. The amplification results showed LRV2 in 7 TR isolates and 2 TR isolates collected from patients. Statistical analysis showed no relationship between response to Glucantime treatment and the presence of LRV2 (*p* value = 0.1086).

No LRV1 was obtained in any sample.

#### Melting curve analysis

For verification of the specific amplification for kmp11 and LRV2, the melting curve analysis was done. The results showed that the amplifications were done specific with the melting temperature of 82.6 °C for kmp11 and 79.8 °C for LVR2.

### Discussion

In this study, 30 CL cases were included, 15 with TF to Glucantime and 15 with TR to Glucantime. All 30 isolates were checked for LRV1 and LRV2, 7 TR isolates and 2 TF isolates showed positive amplification for LRV2. Every clinical isolate was negative for LRV1. Various modalities are used to treat CL [22], World Health Organization standard recommended treatment is to use pentavalent antimoniate derivatives, but a portion of CL patients do not respond to this chemotherapy [23–25]. There are a few reports about the relation of *Leishmania* RNA virus infection and TF [26, 27]. It was previously demonstrated that presence of LRV1 may affect the *Leishmania* virulence [28]. According to Pereira et al. [21] study, there is no relationship between LRV and *L. braziliensis* response to treatment, which agreed with the results of the current study. The study by Hajjaran et al. [8] showed that not only LRV2 inside the *Leishmania* causative agents of CL, also LRV inside the causative agent of kala-azar have no relation with response to treatment similar to the current study. On the contrary, some studies showed a significant relationship between the presence of LRV1 and TF [6, 7, 27].

Hartley et al. [17], Parmentier et al. [28], and Ives et al. [19] showed resistance patterns to chemotherapy in *Leishmania* isolates infected with LRV1, that the presence of LRV1 affects the inflammatory responses and parasites metastatic behavior. In particular, LRV1 in *L. guyanensis* activates strong immunogenicity and triggers host immune system inflammatory factors [25]. The consequences of the last event are related to a more acute form of the disease and also resistance to chemotherapy. The effect of LRV1 on immune response is via toll-like receptor 3 (TLR3) and activation of pro-inflammatory factors and chemokines and therefore increases tissue damages [29]. In the present study, the TF isolates showed no infection with LRV1 but two TF isolates showed to be infected with LRV2, suggesting that other mechanisms might be related to TF. Cantanhêde et al. [18] found that LRV1 is one of the most important factors associated with exacerbation of the lesion and increases the risk of mucosal involvment.

## Limitations

The results of the current study suggested no relationship between *L. major* infected with LRV2 and response to treatment although the sample size is small and further studies needed to clarify the role of LRV in response to treatment.

## Supplementary information

**Additional file 1: Figure S1.** Agarose gel electrophoresis for molecular detection of *Leishmania* genus using L5.8 s and LITSR primer pairs. Line 1: 50 bp DNA ladder, line 2: negative control, line 3: positive control: *L. major* (MRHO/IR/75/ER), lines 4 and 5: clinical isolates with *Leishmania* genus. The expected fragment was around 300–350 bp for *Leishmania* spp. detection.

**Additional file 2: Figure S2.** Agarose gel electrophoresis for RFLP analysis. Line 1: 50 bp DNA ladder, line 2: positive control: *L. major* (MRHO/IR/75/ER), lines 3 and 4: clinical isolates of *L. major*. The fragments with the size of 220 and 127 bp was considered as *L. major.*

## Data Availability

All data generated or analyzed during this study are included in this published article.

## Abbreviations

CL: Cutaeous Leishmaniasis
LRV: Leishmania RNA virus
TF: Treatment failure
TR: Treatment response
