# Supplementary figures and images for: Relationship of Leishmania RNA Virus (LRV) and treatment failure in clinical isolates of Leishmania major
Source: BMC Res Notes. 2020 Mar 4;13:126. doi: 10.1186/s13104-020-04973-y (PMC7074996; doi:10.1186/s13104-020-04973-y)

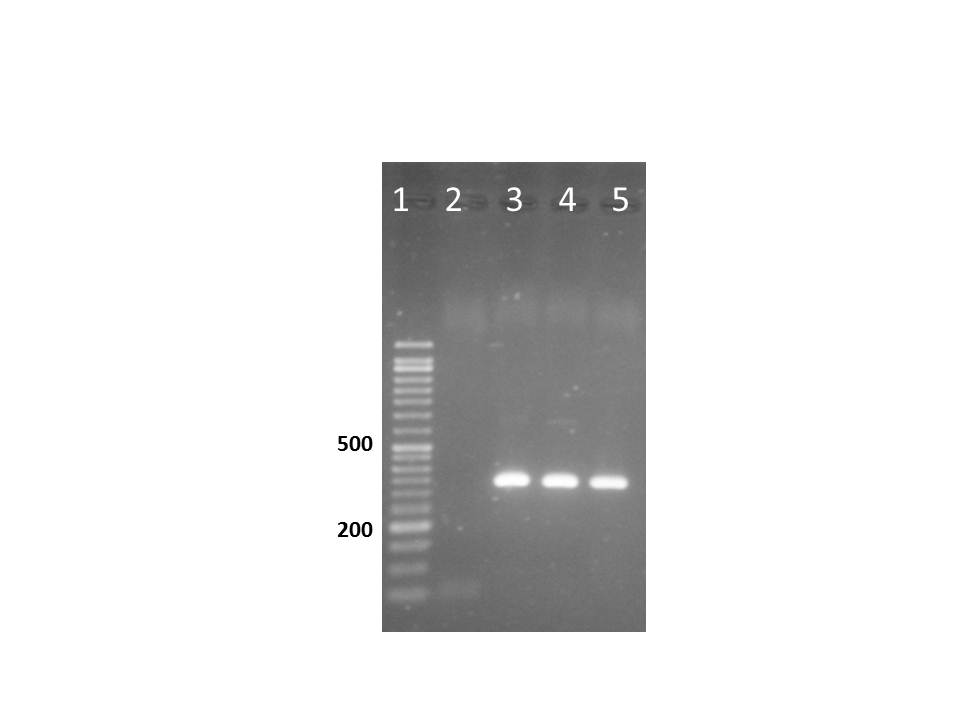

Supplement: Supplementary file 1 — Additional file 1: Figure S1. Agarose gel electrophoresis for molecular detection of Leishmania genus using L5.8 s and LITSR primer pairs. Line 1: 50 bp DNA ladder, line 2: negative control, line 3: positive control: L. major (MRHO/IR/75/ER), lines 4 and 5: clinical isolates with Leishmania genus. The expected fragment was around 300–350 bp for Leishmania spp. detection. [file 13104_2020_4973_MOESM1_ESM.jpg]

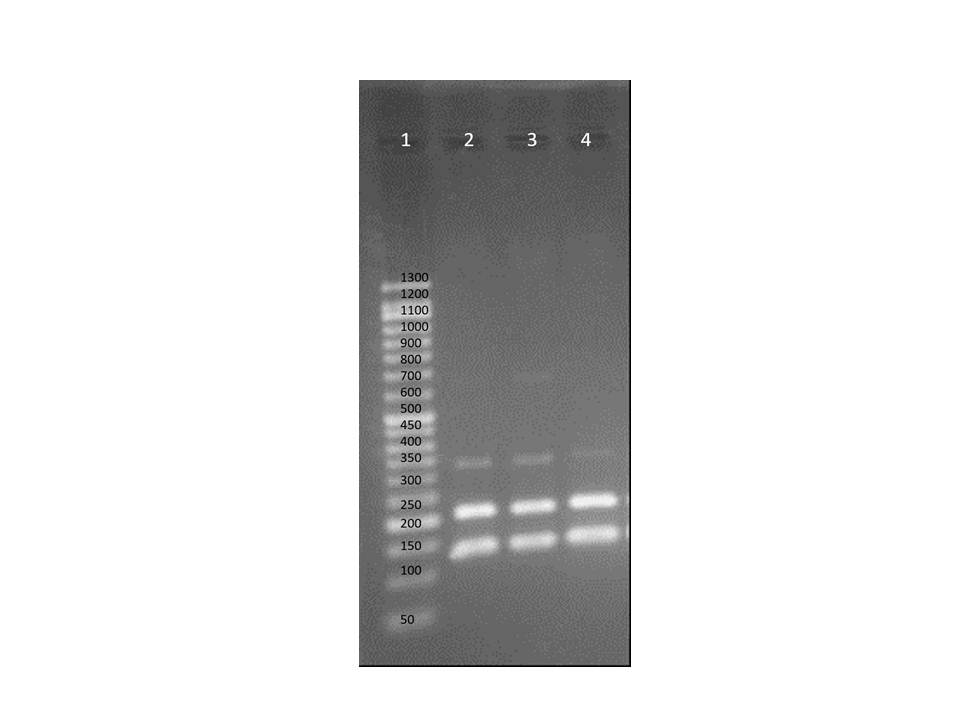

Supplement: Supplementary file 2 — Additional file 2: Figure S2. Agarose gel electrophoresis for RFLP analysis. Line 1: 50 bp DNA ladder, line 2: positive control: L. major (MRHO/IR/75/ER), lines 3 and 4: clinical isolates of L. major. The fragments with the size of 220 and 127 bp was considered as L. major. [file 13104_2020_4973_MOESM2_ESM.jpg]
